# Editorial Comment: The effect of low-intensity shock wave therapy on moderate erectile dysfunction: A double-blind, randomized, sham-controlled clinical trial

**DOI:** 10.1590/S1677-5538.IBJU.2023.01.05

**Published:** 2023-01-26

**Authors:** Rodrigo R. Vieiralves

**Affiliations:** 1 Hospital Federal da Lagoa Serviço de Urologia Rio de Janeiro RJ Brasil Serviço de Urologia, Hospital Federal da Lagoa, Rio de Janeiro, RJ, Brasil

Dimitrios Kalyvianakis^1, 2^, Ioannis Mykoniatis^1, 2^, Nikolaos Pyrgidis^2^, Paraskeui Kapoteli^1^, Filimon Zilotis^1^, Agrippina Fournaraki^1^, Dimitrios Hatzichristou^1, 2^

**J Urol. 2022 Aug;208(2):388-395.**

**DOI: 10.1097/JU.0000000000002684 | ACCESS: 35830338**

## COMMENT

The undeniably numerous advances in the treatment of erectile dysfunction (ED) over the last two decades have not been able to satisfy the desire to search for a treatment modality capable of altering the underlying pathophysiology of the erectile mechanism. The search for a new modality of clinical treatment that is effective with a lasting effect that restores spontaneous erection has brought the focus of recent studies to a promising modality through low-intensity shock wave therapy (LiST), being treated as one of the treatments that has been increasingly proposed.

In 2010, when Vardi et al. first reported the use of LiST for ED (1), the proposed treatment has proven to produce structural changes that regenerate penile tissue, resulting from focused shock waves that interact with the target tissue, induced mechanical stress, and local microtrauma, generating a response with local angiogenic mediators that ultimately promote neovascularization of affected tissues and increase blood flow. The use in vasculogenic ED is postulated as the only currently marketed treatment that can offer a cure, which is the most desired outcome for most men suffering from ED, as outlined in the European Society of Urology guidelines. (2–5).

Despite the promising scenario, prospective randomized studies are scarce, conflicting with many unresolved questions. Furthermore, several aspects make it difficult to perform qualitative studies, such as the heterogeneity among shock wave generators, the type of shock waves emitted, and treatment protocols (duration of treatment, number of sessions per week, total number of delivered shock wave pulses, and penile application sites).

This very interesting article by Kalyvianakis et al., (6) offers another step in our long journey ahead. Through a double-blind, randomized clinical trial controlled by Sham, the effect of Low Intensity Shockwave Therapy on moderate erectile dysfunction was evaluated.

Seventy men aged 40 to 70 years, in heterosexual relationships, stable for more than 3 months, with clinically diagnosed erectile dysfunction, were randomized to 12 sessions of LiST or sham therapy (35 patients) twice a week. Patients were evaluated 1 and 3 months after the end of treatment. The results of the present study suggest that LiST is effective and safe for moderate vasculogenic erectile dysfunction as more than two-thirds of patients showed significant improvement on the IIEF-EF scale.

However, some relevant aspects need to be highlighted. The assessment of vasculogenic ED, one of the inclusion criteria, was defined by clinical criteria that were not clearly described in the present study. The lack of objective data on the presence of vasculogenic erectile dysfunction such as the penile doppler or penile elastography, or even the description of underlying comorbidities, overshadows the underlying clinical condition. Stratifying the results according to comorbidities (hypertension, diabetes, obesity, dyslipidemia, etc), observing groups with more severe comorbidities, independently of the IIEF, could allow us to observe an association between severe pathologies (greater damage to the corpora cavernosa) and negative impact on the effect of LiST (7–9).

Moreover, regarding the application technique, no figure was described or presented application points (penile shaft, crura), making it difficult to understand the standardization of the method, in addition to no report if the treatment was performed by a single applicator or even if there was some prior training.

Corroborating with previous observations, phosphodiesterase type 5 (PDE5) inhibitor or other erectile dysfunction treatments were prohibited during the entire treatment / follow-up period. The strategy to eliminate the bias of an effect of PDE5 inhibitors is interesting, but the question remains: could this class of drugs optimize the local action of shock wave therapy? Future studies using this class of drugs versus placebo would be interesting to rule out this hypothesis.

Other limitations of the present study, appropriately mentioned by the authors, are the profile of patients from a single institution, the total n (70) and the profile of patients - exclusively with moderate ED. Furthermore, the short follow-up period (1 and 3 months) precludes conclusions regarding durability and peak of effect.

To conclude, we see in this important article one more confirmation of the effect of LiST, highlighting the importance of advancing in this field. It seems that LiST is here to stay, perhaps not as a major turning point in the treatment of ED but as a good adjuvant therapy applicable to a selected group of patients, capable of influencing and modifying outcomes.
